# Valorisation of Underutilized Grass Fibre (Stem) as a Potential Material for Paper Production

**DOI:** 10.3390/polym14235203

**Published:** 2022-11-29

**Authors:** Chuan Li Lee, Kit Ling Chin, Paik San H’ng, Mohd Sahfani Hafizuddin, Pui San Khoo

**Affiliations:** 1 Institute of Tropical Forestry and Forest Products, Universiti Putra Malaysia, Serdang 43400, Malaysia; 2 Faculty of Forestry and Environment, Universiti Putra Malaysia, Serdang 43400, Malaysia; 3 Centre for Advanced Composite Materials, Universiti Teknologi Malaysia, Johor Bahru 81310, Malaysia

**Keywords:** Napier grass, sugarcane, grass fibre, chemical pulping, mechanical pulping, alkaline pre-treatment, beating

## Abstract

An integrated and feasible approach was proposed using the underutilized grass fibre (stem) derived from Napier grass and sugarcane for paper production in this study. To enhance paper strength, pre-hydrolysis and beating techniques have been used to improve the chemical pulps and mechanical pulping process, respectively. Napier grass and sugarcane are promising non-wood sources for pulp production, owing to their high cellulose and low lignin and extractive content. With the additional mild alkaline pre-treatment to the mechanical pulping process, the lignin content was greatly reduced. The results reveal that the mechanical pulping with alkaline pre-treatment may indeed potentially replace the most prevalent pulping process (chemical pulping). As evidenced by the paper strength properties, mechanical pulping is far more suitable for grass-type biomass, particularly Napier grass, which had a folding endurance capability five times greater than chemical pulping. Furthermore, the remaining high hemicellulose content from mechanical pulping contributed to a high pulp yield, while also facilitating the fibrillation on the sugarcane’s laboratory paper handsheet. The findings also demonstrated that the additional beating process from chemical pulping causes the fibres to be drawn toward each other, resulting in a more robust fibre network that contributes to good paper strength. Consequently, this work sheds new light on the development of advanced paper derived from grass fibre.

## 1. Introduction

Grasslands are among the most common types of covered vegetation, accounting for more than 31.5% of the world’s land mass. It is one of the sustainable resources and provides a significant portion of livestock with a food source [1]. Among all the grasses, Napier grass (*Pennisetum purpureum*) is a fast-growing monocot grass in the *Poaceae* family of which is morphologically stout, tall, and a deep-rooted perennial bunchgrass, primarily propagated by stem cutting [2,3,4]. Owing to its high productivity and nutritive value, Napier grass is currently the most popular fodder grass in dairy and feedlot production systems in Malaysia [5,6]. This grass is productive, easy to cultivate, drought-tolerant, and adaptable to a variety of soil and climatic conditions, particularly in tropical soils. The wildlife department of Sabah state even cultivated Napier grass for grazing as part of efforts to minimize human–animal conflict and crop destruction [7]. Given its high cellulose content, Napier grass has the potential to produce biofuels such as alcohol, ethanol, butanol, and methane [8,9]. In addition, another fascinating grass which is currently used as primary raw material for biofuel products is the giant grass known as sugarcane (*Saccharum officinarum*). Sugarcane is distinct from other types of grass in that it can store vast amounts of sugar as sweet juice in its fibrous stalk/stem. Sugarcane ethanol is produced from sugarcane fermentation and can effectively be used as an ethanol-gasoline blend or as a 100% pure ethanol fuel in spark ignition engine vehicles. The favourable climate and agricultural capabilities of Malaysia enable the production of a high yield of sugarcane, which opens up the possibility of an alternative fuel source [10].

Aside from the biofuel implementation programme, Malaysia is in an urge to start introducing green and sustainable pulping processes, and grass fibre-based paper products may sound promising. In recent years, there have been some major breakthroughs in Malaysia’s papermaking industry with the use of oil palm biomass in two notifiable companies producing pulp and paper products from empty fruit bunch fibres [11,12]. However, Malaysia is still highly dependent on the imports of pulp to meet the domestic demand of paper products. Wood is the primary source of cellulose fibre used in the paper industry in Malaysia, with 80% of the virgin pulp imported [13]. Due to a recent global shortage of woody products, pulp supplies have been one of the major issues in the paper industry [14]. Thus, the use of non-wood fibres that can be processed into pulp with less chemicals or energy input is a promising cost-effective strategy in producing pulp and paper. Napier grass and sugarcane have two primary parts in terms of structure—the leaf and the stem. Due to its high protein content, nutrition, and desirable texture, the Napier grass leaf is suitable for use as animal fodder (e.g., softness). The stem, on the other hand, is underutilized due to its hardness, high density, and high lignin content [15,16]. In the case of sugarcane, the dry pulpy residue left after extracting juice from the stem was also underutilized as well. The valorisation of all this underutilized biomass via a pulping process could be the solution to an enhanced biomass conversion.

Paper pulp can be divided into two types based on the pulping method used: chemical pulp and mechanical pulp. Chemical pulp is made using the chemical pulping process, whereas mechanical pulp is made using the mechanical pulping process. Mechanical pulping is appealing to pulp producers because it is less expensive and easier to implement than chemical pulping [17]. To improve the efficacy of fibrillation, the fibre may be pre-softened by heat prior to mechanical pulping, known as thermo-mechanical pulping. Despite the fact that thermo-mechanical pulping is a mainstay of the pulping industry, thermo-mechanical pulping mills face a number of challenges as a result of rising electricity costs and a push to reduce greenhouse gas emissions [18]. The pulp and paper industry is one of the world’s largest users of energy and water. The paper and pulp industry was reported as the fourth largest emitter of greenhouse gases in the manufacturing sector of the United States of America. It was also reported in India that this industry produces approximately 100 million kg of hazardous pollutants annually [19]. Common solid pollutants include wood waste, sodium salts from the recovery boiler, pulp screening rejects, dregs, and grit from causticizing plants [20,21]. Researchers in the pulp and paper sector are always in an effort to improve the pulping technique, with an emphasis of not only to replace wood resources as a raw material, but also to provide insights into environmental practices that can move beyond the conventional pulping methods.

Malaysia’s paper consumption is growing steadily, putting a large and constant demand on the supply of fibres for pulp and paper production. At this juncture, with the fascinating characteristics such as abundance volume, a short cycle growth, and environmental friendliness, grass fibres may act as a great alternative to replace wood fibres. We believe that the advanced pulping technique will enable grass-type fibre to be a game changer in the pulp and paper industry. To fully comprehend the potential of grass fibre (stem) for pulp and paper production, chemical and mechanical pulping were applied to two grass-type biomasses, Napier grass and sugarcane stem. The characteristics for both grass fibres’ laboratory paper handsheets produced using varying pulping techniques were also evaluated. The strength of the paper made from grass fibre presents another challenge in the production of paper. Pre-hydrolysis techniques and beating techniques have been used to improve chemical pulps and the mechanical pulping process, respectively. Thus, this research will also investigate into the efficacy of pulp and fibre modification via the beating process on chemical pulping and alkaline pre-treatment on mechanical pulping.

## 2. Materials and Methods

### 2.1. Raw Materials Preparation

Samples of Napier grass and sugarcane were supplied by Regu Yakin Sdn. Bhd. (Pahang, Malaysia). Both plants were propagated vegetatively through stem cuttings in Pahang. The Napier grass and sugarcane were harvested after 4 and 6 months, respectively. The harvested Napier grass stems were washed before being dried in a forced-air drying oven at 60 °C overnight, whereas the sugarcane stems were collected and dried after the sugar extraction process. The dried stems were then chipped using a chipper machine (PZ 8, Pallmann Maschinenfabrik GmbH & Co, Zweibrücken, Germany) and screened to a width of approximately 2 cm to enable better chemical penetration during pulping. The samples were then air-dried to 10% moisture content (storage) prior to pre-treatment.

### 2.2. Pulping Process

#### 2.2.1. Mechanical Pulping

The fibres were pre-treated by being fully immersed in a 3% sodium hydroxide (NaOH) solution with a solid-to-liquid ratio of 1:10. The impregnation process was held for 24 h. The fibres were then washed with tap water for approximately 30 min, or until the soapy condition diminished. The Refiner Mechanical Pulper (RMP) machine (Andritz Sprout Bauer, Muncy, PA, USA) with 1/65-inch disc gap was used to refine the washed fibres. The refining process was carried out with the disc plate gaps set at 2.5 mm between the refining plates. The two-cycle refining process was designed to reduce the negative impact of the plates’ harsh actions on the fibres. The wet fibres were manually squeezed after refining to remove the water.

#### 2.2.2. Chemical Pulping (Kraft Pulping)

Table 1 summarises the chemical pulping conditions for Napier grass and sugarcane stem. The chemical pulping was carried out using Twin Digester (MK Twin Tub Digester, model GTD-15L, GIST Co Ltd., Korea). Both mechanical and chemical pulping machines were provided by the Institute of Tropical Forestry and Forest Products (INTROP), Universiti Putra Malaysia, Selangor, Malaysia. Notably, the entire chemical pulping process was in a closed system.

### 2.3. Screening

Prior to screening, these pulps were dispersed in a Hydropulper (Sheng Feng, Henan, China). The pulps were screened using PTI Sommerville Fractionators with 0.30 mm slots. The oversized debris particles from the stem pulp were removed via screening.

### 2.4. Spin Drying

Both types of pulps were spin-dried to remove residual water using a spinning machine. The screened pulps were spin-dried for 5–10 min in a fabric bag. After the spinning process was completed, the pulps were weighed to determine the pulp yield percentage. Finally, the pulp was stored in a freezer at 6 °C. A specific weight of the pulp was placed in an oven to dry at 105 °C overnight to establish the oven-dry mass. The screened pulp yield was calculated using the formula:

(1) $$\mathrm{Pulp}\;\mathrm{yield}\;(\%)=\frac{Oven\;dry\;mass\;of\;pulp}{Initial\;oven\;dry\;mass\;of\;wood\;chips}\times 100$$

### 2.5. Laboratory Handsheet Making Process

Beaten samples were prepared in accordance with standard condition TAPPI T 248 sp-00 [22]. A total of 500 revolutions were used as the beating degree. A specified mass of Napier grass and sugarcane (beaten and unbeaten pulp) were fiberized in a disintegrator at room temperature and 10% consistency for 20 min using a laboratory PFI mill machine. Finally, laboratory paper handsheet production was conducted using a laboratory handsheet machine based on the TAPPI Standard, T 205 sp-02 [23] to produce 60 g/m^2^ of laboratory paper handsheets. Before any evaluation, these laboratory paper handsheets were conditioned for 24 h at a temperature of 23 °C ± 1 °C and 50% ± 2% relative humidity.

### 2.6. Evaluation

#### 2.6.1. Chemical Composition

The chemical composition of the raw materials and produced pulps from Napier grass and sugarcane stems were determined according to TAPPI standard. All experiments were carried out in five replicates to obtain an accurate result.

##### Extractives

The extractive content of the sample was determined according to TAPPI T 204 cm-97 standard. The ethanol-acetone solution was prepared by a mixture of approximately 95% ethyl alcohol and acetone as a reagent in the ratio of 3:1. A sufficient amount of the sample equivalent to 2 g of the powder of CS and PKS was weighted separately and placed into the thimble. The thimble with the sample was then placed in a dry Soxhlet extraction apparatus. The extraction flasks were then filled with 200 mL of the ethanol-acetone solution. The flasks were connected to the extraction apparatus with water flowing to the condenser section. The heater was set to 100 °C and the boiling process took around 4 to 5 h. After the extraction process, the solution was dried using a desiccator and further dried in an oven for 24 h before the extraction content was calculated. The percentage of extractive in lignocellulosic biomasses was calculated as follows:

(2) $$\%\;\mathrm{of}\;\mathrm{Extractive}=\frac{Weight\;of\;flask\left(g\right)+Extraction-Weight\;of\;flask\;\left(g\right)}{ODW\;sample\left(g\right)}\times 100$$

(3) $$ODW=\frac{Weight\;of\;air\;dried\;sample\left(g\right)-Weight\;of\;crucible\left(g\right)}{100}$$

##### Lignin Content

The lignin content of samples was determined according to TAPPI T 222 om-98 standard. Approximately 1 g of oven-dried extraction free sample (Napier grass and sugarcane stem) was added into a beaker with 2% H_2_SO_4_. The mixture was occasionally stirred in a water bath at room temperature and rested for 2 h to ensure a complete dissolution occurred. The solution was then added into distilled water until the total volume of 575 mL. The mixture was then boiled for 4 h by maintaining a constant volume using a reflux condenser. After boiling, the solution was filtered with the aid of a suction machine and washed using hot water. The lignin residue was dried for 1 h at 110 °C and then allowed to cool in a desiccator. The percentage of lignin was calculated as follows:

(4) $$\%\;\mathrm{of}\;\mathrm{Lignin}=\frac{W_{4}-W_{3}}{100\times W_{2}}\times \left(100-W_{1}\right)$$

where,

W_1_ = alcohol-acetone extractive content, %;
W_2_ = weight the of oven-dried extractive-free sample, g;
W_3_ = weight of oven-dried crucible, g;
W_4_ = weight of oven-dried residue and crucible, g.

##### Holocellulose

A total of 2 g of the residue from the ethanol-acetone extraction was placed into a 250 mL beaker and closed with a watch glass. The specimen was added with 100 mL of distilled water, 1.5 g of sodium chlorite, and 5 mL of 10% acetic acid. The beaker was placed in a hot water bath or on a hot plate maintained at 70 °C; the contents were swirled vigorously once every 5 min. The flask was kept closed with a small, inverted Erlenmeyer flask. The whole experiment was carried out in a fume hood. After 30 min, 5 mL of 10% acetic acid was added. After 30 min, 1.5 g of sodium chlorite was additionally added on and acetic acid and sodium chlorite were continued until 6 g of sodium chlorite was incorporated in the solution. Then, the mixture was heated for 30 min after the last addition of sodium chlorite. The suspension was cooled in an ice bath. It was filtered into a weighed fruited glass crucible and washed with iced distilled water. Finally, it was washed with acetone. The residue was air dried for one to two days until it was free of acetone and covered by perforated aluminium foil. It was transferred to a desiccator and weighed at daily intervals until the sample reached a constant weight. The percentage of holocellulose was calculated as follows:

(5) $$\%\;\mathrm{of}\;\mathrm{Holocellulose}=\frac{W_{4}-W_{3}}{100\times W_{2}}\times \left(100-W_{1}\right)$$

where,

W_1_ = alcohol-acetone extractive content, %;
W_2_ = weight of oven-dried extractive-free sample, g;
W_3_ = weight of oven-dried crucible, g;
W_4_ = weight of oven-dried residue and crucible, g.

##### Alpha-Cellulose

The cellulose content in both samples were carried out according to TAPPI 203 standard using 8.3 and 17.5% NaOH and 2 N of acetic acid (CH_3_CO_2_H). A total of 2 g of the sample was put into a 250 mL beaker. Then, 15 mL of 17.5% NaOH was added and gently macerated with a flattened glass rod for 1 min. A total of 10 mL NaOH was further added into the solution and stirred for 45 s, then 10 mL of NaOH was added and stirred for 15. The mixture was stirred and allowed to stand for another 3 min. A total of 10 mL of NaOH was added and mixed with a stirring rod every 2½ min. These steps were repeated for three times until the total time reached 15 min. The beaker was covered with a watch glass and the mixture was left in a water bath for 30 min. Then, 100 mL of distilled water was added quickly at 20 °C and thoroughly mixed, and the diluted mixture was left in the water bath for a further 30 min. The mixture was filtered into a weighed fruited glass crucible with coarse porosity. The beaker and residue were rinsed with 25 mL of 8.3% NaOH and 650 mL of distilled water. Then, the residue was rinsed with 2N CH_3_CO_2_H and rinsed again with distilled water. The crucible was placed in the oven at 105 °C. The α-cellulose was calculated as a percentage, as shown in Equation (5). The amount of hemicellulose was calculated by subtracting alpha-cellulose from holocellulose.

(6) $$\%\;\mathrm{of}\;\mathrm{Alpha}-\mathrm{cellulose}=\frac{W_{4}-W_{3}}{100\times W_{2}}\times W_{1}$$

where,

W_1_ = Holocellulose content, %;
W_2_ = weight of oven-dried holocellulose sample, g;
W_3_ = weight of oven-dried crucible, g;
W_4_ = weight of oven-dried residue and crucible, g.

#### 2.6.2. Laboratory Handsheet Properties Determination

Laboratory paper handsheets were examined in terms of their physical, mechanical, and optical properties. To ensure the uniform distribution of Napier and sugarcane stem fibres, the pulp slurry was disintegrated using a Pulp Disintegrator (Regmed DSG 200) at 25000 rpm before the production of laboratory paper handsheets. All results in this study were expressed as mean ± standard deviation of three replicates. The sample was prepared with the required testing size in accordance with the relevant standard, and the following parameters were evaluated:

(a) The bursting test was performed by bursting strength tester (GT-7013-ADP) according to TAPPI T403 om-02 [24].
(b) The opacity test was performed by Technidyne Brightimeter (Micro S-5) according to TAPPI Standard, T 425 om-01 [25].
(c) The brightness test was performed by Technidyne Brightimeter (Micro S-5) according to TAPPI T425 Om-02 [26].
(d) The tensile test was performed by a universal testing machine (UTM Twin Column Lyod 2.5 kN) according to TAPPI Standard, T 494 om-01 [27].
(e) The tearing test was performed by Elmendorf ProTear Tester (Thwing-Albert) according to TAPPI Standard, T 414 om-98 [28].
(f) A morphological observation of the laboratory paper handsheet.
(g) The morphological properties of handsheets were observed via Scanning Electron Microscopy (SEM) (Hitachi S-3400N and Jeol JXA 840A) under an accelerating voltage of 15 kV. Before scanning, samples were coated with gold using a sputter coater system (Edwards Sputter Coater; BOC Edwards, Crawley, West Sussex, UK) to obtain an excellent image by avoiding any charging effect.

### 2.7. Statistical Analysis

Statistical analyses were conducted using the statistical package SPSS for Windows, version 16.0 (SPSS, Chicago, IL, USA), which was used to evaluate the adsorption property data of the laboratory paper handsheet properties for analysis of variance (ANOVA) at a 95% confident level (*p* ≤ 0.05). Results were analysed using one-way ANOVA, followed by Tukey’s test as a post hoc test [29]. The Tukey–Kramer multiple comparisons test was applied to analyse the differences between the treatment effects when significance was observed. The effects were considered to be not statistically significant when the *p*-value was higher than 0.05 at the 95% confidence level.

## 3. Result and Discussion

### 3.1. Chemical Composition Analysis

Table 2 summarizes the contents of cell wall structural constituents (cellulose, hemicelluloses, and lignin) in Napier grass, sugarcane biomass, and pulp obtained. Despite their high ash content, both biomasses are ideal for pulping, owing to their low lignin content. According to Kontturi et al. [30], cellulose is the primary structural component of plant cells. As shown in Table 1, both biomasses have a high α-cellulose content, which has the potential to result in high pulp fibre yields. Sugarcane contains a higher ash content than Napier grass (refer to Table 1). The use of non-wood as a pulping raw material in conventional processes is limited by the presence of high silica levels [31]. The chemical analysis also revealed that both biomasses had a low extractive content. Materials with a low extractive value are more likely to generate a high yield during the cooking process [32]. A chemical composition comparison reveals a significant difference between the raw fibre and the obtained pulp. The obtained pulp contained more cellulose, while other content, particularly lignin content, was highly removed. The disruption of lignin–carbohydrate complex (lignin-hemicelluloses) linkages during the alkaline treatment significantly reduced the lignin and hemicellulose content [33] from both of the grass-type fibres.

Undoubtedly, a higher lignin content necessitates a large amount of energy and chemicals [34]. The presence of lignin is detrimental to the stability of chemical and mechanical paper properties [35]. It was even necessary to bleach for some applications. The lignin content of Napier grass is 21.77% and that of sugarcane is 24.32%, both of which are low, implying that both biomasses ought to be fairly easy to pulp than wood with a lignin content of 26–30%. Furthermore, biomass fibre with a low lignin content is easier to delignified and requires milder and faster cooking conditions [36]. With the additional mild NaOH pre-treatment to the mechanical pulping process, the lignin content was greatly reduced. Ultimately, the high cellulose and low lignin and extractive content of non-wood resources of Napier grass and sugarcane stem qualify them as promising raw materials for pulp production. This result is benefitted by using low-cost biomass and, more notably, environmentally benign pulping processes (with mild chemical usage from the mechanical pulping process), which could be the new market trend for the pulping industry.

### 3.2. Yield of Pulp

**Figure 1:**
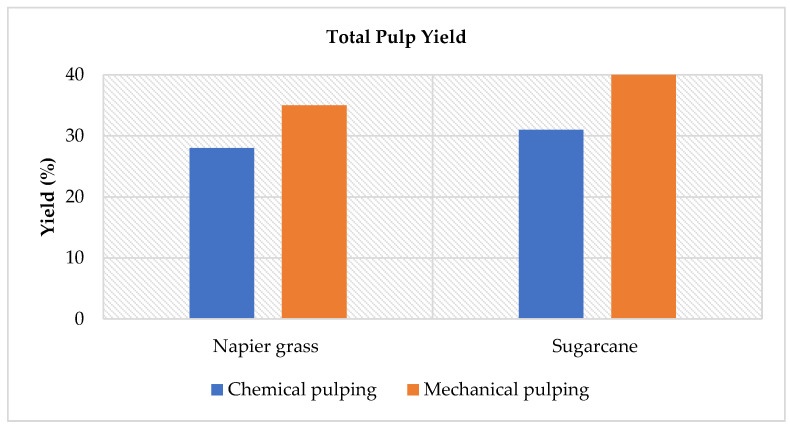
Pulp yield of the Napier grass and sugarcane stem.

The pulp yield for Napier grass and sugarcane stem are illustrated in Figure 1. Sugarcane has a higher yield than Napier grass due to the presence of more hemicelluloses. Higher hemicellulose content appears to improve the pulp yield [37]. Xylan, which makes up approximately 24–25% of the main hemicellulose in the sugarcane holocellulose structures, is important because it contributes to increase pulp yield [38]. In comparison to chemical pulping, mechanical pulping offers a higher pulp yield. Table 2 shows that the chemical pulping process led to a significant delignification effect on both samples. The removal of lignin and carbohydrates may lead to a significant decrease in pulp yield [39]. Since a higher yield was obtained, it is evident that mechanical pulping is preferable as it will increase the producer’s profit margin.

Mechanical pulping uses less chemicals than chemical pulping. Chemical activity devalues and solubilizes biomass components, primarily lignin and hemicellulose, resulting in lower pulp yield and lignin content [40]. Furthermore, chemical pulping necessitated the recovery of chemicals from spent cooking liquor, the recovery of heat energy from the heating of recovered lignin and other organic materials from the black liquor, and the reduction in air and water pollution [41]. The mechanical pulping process has been demonstrated to be a more environmentally friendly method that helps to produce a laboratory paper handsheet that degrades naturally and lowers the levels of pollution worldwide through this study.

### 3.3. Mechanical Properties

Table 3 summarizes the laboratory handsheet properties derived from Napier grass and sugarcane stem. The Tukey–Kramer multiple comparisons test was applied to analyse the differences between the treatment effects when significance was observed. The ANOVA analysis revealed significant effects (*p* < 0.01) for the type of biomass, pulping technique, and the interaction of the two factors.

Table 3 depicts the mechanical properties of a laboratory paper handsheet made from Napier grass and sugarcane stem using various pulping techniques. This study found that a laboratory paper handsheet derived from mechanical pulping outperforms chemical pulping in terms of folding endurance. Folding endurance is an empirical test that determines how many folds a piece of laboratory paper handsheet can withstand before its tensile strength drops below a certain level [42]. The result reveals that mechanical pulping remarkably enhanced the folding endurance of the laboratory paper handsheets from these two types of biomass, particularly Napier grass, for which mechanical pulping is five times better than chemical pulping. Throughout, in regard to folding properties, chemical pulping had lower tensile, tear, and burst indexes than mechanical pulping.

Chemical pulping was the most widely used method in the pulp and paper industry. However, the currently used chemical pulping procedures are primarily adapted from wood pulping; with this procedure, the operating mills face difficulties accepting other raw materials such as grass-type biomass [43]. Many published researches have stated the distinct advantages of mechanical and chemical pulping, with mechanical pulp providing paper with higher bulk properties, while chemical pulp provides paper with higher tensile stiffness [44]. Nonetheless, the results from this study show that the tensile strength of both grass-type laboratory paper handsheets prepared by chemical pulping is extremely low compared to laboratory paper handsheets from mechanical pulping. The tensile strength of laboratory paper handsheets derived from chemical pulping was increased with the additional beating process. With an additional beating treatment, the tensile strength of laboratory paper handsheets derived from chemical pulping was doubled. This could be due to the fact that tensile strength is primarily affected by inter-fibre bonding force and fibre strength, and beating clearly promoted inter-fibre bonding [45,46]. Furthermore, improved fibrillation induced by the beating process may also lead to improved fibre conformability, resulting in a stronger fibre network structure [47].

The results demonstrate that laboratory paper handsheets made from both unbeaten pulps have a low tearing resistance. Unbeaten pulp commonly has poor inter-fibre contacts during the laboratory paper handsheet formation, which results in poor laboratory paper handsheet properties. Beaten pulps have highly improved the mechanical properties of laboratory paper handsheet specimens significantly. The enhancement is also ascribed to the increase in the bonded area of the sheet, resulting from internal and external fibrillation that occurs during beating. Inter-fibre bonding is crucial to sheet strength because it is determined by two factors: the strength of an individual fibre and the inter-fibre bonds. They make a significant contribution to the internal cohesion of paper. As inter-fibre bonding strengthens, it is more likely that a fibre in the path of an advancing tear will be severed rather than pulled out. Additionally, all the while, stress is concentrated at the apex of the tear and is challenging to share with the rest of the structure [44,48,49]. Defibrillation and delamination occur during the beating process, resulting in an increased fibre surface area, improved contact quality, and flexibility [50]. Respectively, fibre flexibility increases the number of bonds required to provide the bonding area, resulting in a larger surface area for bonding [51,52]. Because of internal and external fibrillation, the development of fibre surfaces for bonding may be enhanced during the beating of chemical pulp. Since the good inter-fibre contact was created by the beating process, the laboratory paper handsheets can withstand more stress before tearing.

The amount of hydrostatic pressure applied to a circular sample area is defined as bursting strength. It indicates the resistance of the laboratory paper handsheet to rupturing, and laboratory paper handsheet with a low burst strength cannot easily retain packed goods and tears [53]. Besides, the bursting strength also determined the capability of packaging when it was subjected to stacking, collapsing, striking, tearing, and squeezing, all of which were greatly improved multiple times in both dry and wet states [54]. This study reveals that fibre obtained directly from pulping is simply inadequate for laboratory paper handsheet production. They must first be refined through the beating process or the alkaline pre-treatment. The burst strength resistance of the laboratory paper handsheets improved with the beating process and mechanical refining process. The knife-edges or bars in the refiner or beater abrade and fibrillate the fibres. Mechanical squeezing and pounding of cellulose fibre allow water to penetrate its structure, causing swelling and making the fibre more flexible. The pulp fibres are separated, crushed, frayed, fibrillated, and cut during refining or beating. They absorb water and expand, becoming more flexible and pliable. Their ability to bond with one another after drying is greatly enhanced, partly due to the modification of the fibre surfaces and partly due to the creation of the new surface area, which increases the fibre-to-fibre bonding during the laboratory paper handsheet-making process [55]. Remarkably, the results indicate that mechanical pulping is best suited for grass-type biomass because it can achieve higher strengths than the most conventional pulping methods, such as chemical pulping for paper production.

### 3.4. Optical Properties

**Figure 2:**
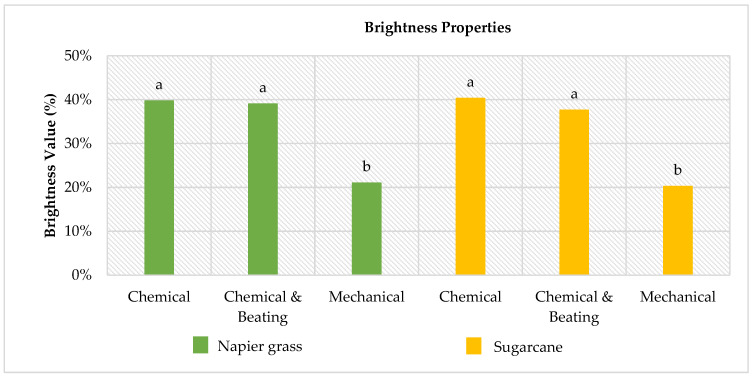
Brightness values of produced laboratory paper handsheets. Means followed by the same letter (a, b) in the same column are not significantly different at *p* ≤ 0.05 according to Tukey’s multiple comparisons test.

**Figure 3:**
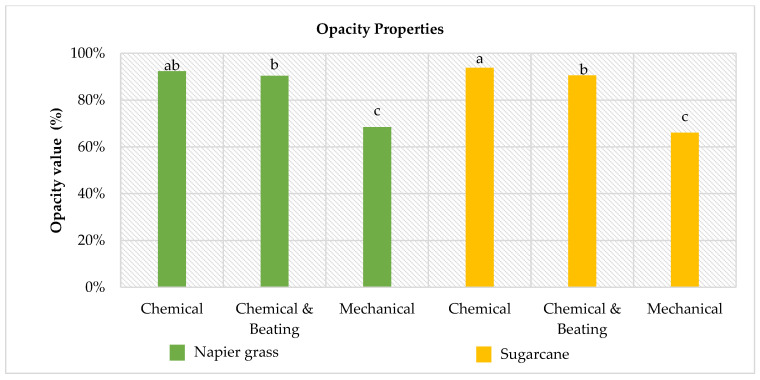
Opacity values of produced laboratory paper handsheets. Means followed by the same letter (a–c) in the same column are not significantly different at *p* ≤ 0.05 according to Tukey’s multiple comparisons test.

Figure 2 and Figure 3 had shown the optical properties (brightness and opacity) of the laboratory handsheet made from Napier grass and sugarcane stem. The Tukey–Kramer multiple comparison test was employed to determine the interaction among the independent variables.

Figure 2 and Figure 3 illustrate the Tukey-Kramer multiple comparisons test, which was used in this study to classify various mean levels of brightness and opacity for six different laboratory paper handsheets. The ANOVA analysis revealed significant effects (*p* < 0.01) for biomass type, pulping technique, and the interaction of the two factors. It clearly shows that the laboratory handsheets made from mechanical pulp had the lowest brightness and opacity. The low value for both opacity tests on the mechanical laboratory paper handsheet was due to the fact that the majority of the original lignin in the raw pulp was still present [56]. The lignin substrate contains many chromophore groups, such as quinoids, catechols, aromatic ketones, stilbenes, conjugated carbonyls with phenolics, and metal complexes, and some additional chromophores within paper can be oxidised from leucochromophores and remnant carbohydrates [57,58,59,60,61]. Chromophores are coloured substances with a high degree of stability that reduce brightness in pulp and fibre [62,63]. The goal of mechanical pulping is to achieve a high yield with high strength properties. Although the lignin may cause the mechanical pulp to turn yellow with exposure to air and light, they are less expensive to produce and yield a higher pulp content [64]. Furthermore, mechanical pulp can be used without bleaching to produce printing papers for applications where low brightness is acceptable, such as newsprint.

A new discovery was made in this current study, as there is a growth in tensile strength but a reduction in the opacity of laboratory paper handsheets from chemical pulping. As stated by [65], laboratory paper handsheets with a high light scattering coefficient indicate small bonding areas. The breaking length, which expresses tensile strength, increases linearly as bond strength per unit area and relative bonded area increase. As a result, enhancing the surface area through the beating process is a crucial method for improving the bonding strength of the fibre and forming a fibrous network. Due to the small bonding area, the opacity degree of laboratory paper handsheets prepared via chemical pulping was reduced. The opacity of Napier grass and sugarcane laboratory paper handsheets was reduced with the additional beating process shown in Figure 3. Figure 3 also shows that the beaten laboratory paper handsheet produced the same brightness as the unbeaten laboratory paper handsheet. As a result, it is concluded that the beating process only affects the opacity properties of the laboratory paper handsheet and not the brightness properties.

### 3.5. Surface Morphology

**Figure 4:**
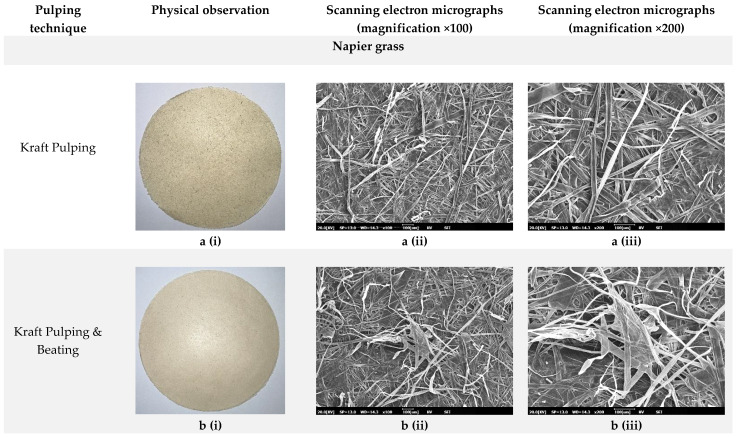

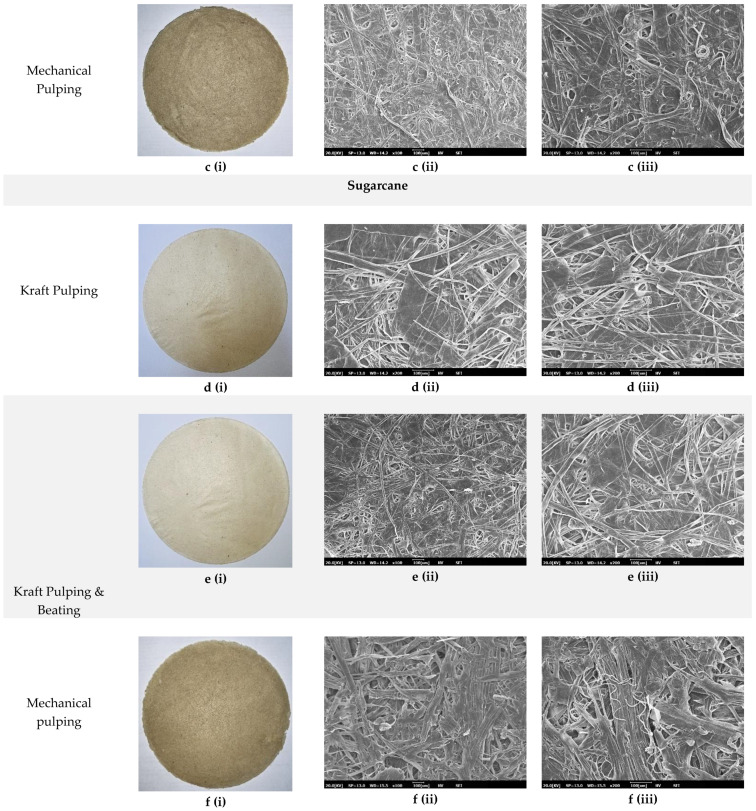
Visual observation and SEM micrographs of produced laboratory paper handsheets.

The visual observation in Figure 4 also shows that beating causes changes in laboratory paper handsheet characteristics. All laboratory paper handsheets produced with beaten pulp fibres were denser than those made with unbeaten pulp fibres. This appears that the pulp fibre has a high felting power but a low flexibility and collapsibility of the fibre wall [66,67]. Through the visual observation, the brightness of the laboratory paper handsheet clearly reflects the efficiency of lignin removal from chemical pulping from both grass-type biomasses. As shown in Figure 2, the laboratory paper handsheet derived from chemical pulping is much brighter as compared to mechanical pulping. The factor that leads to the change in brightness of papers is the presence of lignin. When lignin content is low, the paper will appear brighter [68]. Despite the fact that the high lignin content was responsible for the low brightness on the laboratory paper handsheet derived from mechanical pulping, it provided an interesting feature. When the Napier grass’ laboratory paper handsheets are pressed and folded by hand, the laboratory paper handsheet prepared by chemical pulping is softer than the laboratory paper handsheet prepared by mechanical pulping. A high removal of lignin, hemicellulose, and cellulose through chemical pulping causes the fibres to become soft and more loosened, and therefore, unsuitable for papermaking. This observation explained why the foldance capability of mechanical laboratory paper handsheets derived from Napier grass is far greater than that of chemical pulping.

To better comprehend the distinctions between the laboratory paper handsheets, this study depicts SEM images of laboratory handsheets from Napier grass and sugarcane. Long-fibre materials, as shown in the SEM image of the laboratory handsheet, were made from Napier grass (Refer Figure 4a). The mechanical properties of the paper are influenced by the length of the fibre. According to Gomes et al. [69], long fibre will improve the mechanical properties of the paper. Long fibre pulps have high folding endurance because they have more fibre joints that are able to form a strong network as well as a larger surface area to form bondings with their length spans and width of the fold line [70,71]. The strength of the fibre itself is affected by the packed arrangement of the fibre matrix on the surface and fibre of Napier grass. Inside the Napier grass, there were many matrix fibres and long fibres that crossed with each other (Figure 4b). In this study, lower tensile and tearing indexes occurred in laboratory paper handsheets produced by chemical pulping. As a result, additional mechanical treatment, including the use of beating, could well be needed to improve the paper properties. Paper with an additional beating process has significantly higher paper strength, as shown in Table 3. The improvement could be attributed to the changes in fibre structure induced by the beating process. The SEM results reveal that the additional beating process causes the fibres to be drawn toward each other, resulting in a more robust fibre network that contributes to good paper strength. The long fibre (Figure 4b(ii)) and abundance fibre (Figure 4b(iii)) from Napier grass pulp has greatly contributed to the folding test value.

Furthermore, pulping process conditions, such as high alkalinity, temperature, and mechanical forces, or a combination of these, can result in non-homogeneous zones in the fibre wall, such as deformations [72]. The surface of the chemical pulp of sugarcane was noticeable with prominent deformation, kinks, and curls (refer to Figure 4d(iii)). It has been reported that an increase in fibre deformations such as curls and kinks reduces tensile strength [47]. Such a deformation was resolved by an additional beating process. After the beating process, large parts of the fibre deformation were removed, and the strength properties were restored to undeformed pulp values [73]. When beaten samples were compared to unbeaten samples, the fibres were straighter and had fewer deformations (refer Figure 4e). Some deformations are known to be straightened during the beating process. The beaten sample’s fibres were found to be flatter than the unbeaten sample. This could be because the fibre wall became more flexible as a result of lumen collapse [74].

In addition to fibre straightening, beating causes fibrillation along the fibre surfaces. Fibrillation, or the exposure of cellulose fibrils, will certainly improve the accessible surface area, enhance fibre-fibre bonding, and ultimately, improve laboratory paper handsheet mechanics [75,76,77]. Aside from the tear index, fibre fibrillation significantly increased all of the strength properties (refer to Table 2). The bonding of fibre became increasingly tight, and the fibrillation became more visible with the additional beating process (Figure 4e). As stated by Jiang et al. [78], over half of the increase in fibre strength is due to beating, which straightens the fibre during the pulping process, removing curls and kinks. Additionally, fibrillation can also be attributed to the removal of lignin and other structural effects [79].

Fibrillation is actually explained as a peeling-off mechanism in shearing refiners; the primary wall and S1 layer are peeled off, exposing the S2 layer to inter-fibre bonding [80]. This result indicates that the refining process of mechanical pulping resulted in fibre fibrillation. A highly fibrous network-like structure composed of cellulose microfibrils is visible in the micrograph for mechanical laboratory paper handsheets (Figure 4c,f). Bunches of fibres in the suspension are squeezed and sheared between the refiner’s working surfaces. As a result of this action, parts of the fibre’s outer layers unravel, resulting in fibrillated fibre surfaces. Furthermore, delamination within the cell wall tends to make the fibres more flexible while they are still wet [81]. Apart from that, the removal of lignin and hemicelluloses by alkaline pre-treatments may also be responsible for the morphological changes in the fibre surface. Aside from contributing to a high pulp yield, hemicellulose has a significant impact on sugarcane surface characteristics. The high hemicellulose content of the sugarcane facilitated fibrillation during the mechanical pulping process, as shown in Figure 4f(iii). This can be explained by hemicelluloses acting as inhibitors of microfibril coalescence, and thus, facilitating fibrillation [7,82].

Apparently, the current research reveals that the mechanical pulping with alkaline pre-treatment could indeed replace the most prevalent pulping process (chemical pulping) in producing pulps from Napier grass and sugarcane. As evidenced by the surface morphology and mechanical properties results, mechanical pulping is far more suitable for the grass-type biomass. Furthermore, the high yield value has proven that mechanical pulping is best suited for commercial scale production. This study discovered that the mechanical pulping process on Napier grass and sugarcane could meet the quantity and quality concerns in paper production. This discovery was extremely plausible for industrial paper production because it does not only make economic sense, but it is also more environmentally conscious for this money-generating and constantly expanding industry.

## 4. Conclusions

This research employed fibre from the underutilized discarded grass-type biomass, Napier grass and sugarcane stem, via a pulping process for enhanced biomass conversion in order to establish a zero-waste environment. The concept of using pre-hydrolysis techniques and beating techniques in conjunction with the chemical and mechanical pulping processes, respectively, opens up new possibilities to improve the paper strength. The low lignin content of Napier grass and sugarcane stem emphasizes that both grass-type biomasses ought to be the new frontier materials for the pulping industry. The introduction of alkaline pre-treatment to the mechanical pulping process not only effectively reduced the lignin content of both biomasses, but it also markedly increased the mechanical properties of the paper. Undoubtedly, chemical pulping is more advanced in terms of optical preference, but the high reduction in hemicellulose has resulted in a low pulp yield, which is an important consideration for the pulp and paper industry. The visual observation reveals that the laboratory paper handsheet produced by chemical pulping is softer than the laboratory paper handsheet produced by mechanical pulping. Moreover, the surface of the chemical pulp derived from sugarcane was noticeable with prominent deformation, kinks, and curls, rendering it unsuitable for papermaking. Additional beating is required to make it more suited for paper production as it causes the fibres to be drawn toward each other, resulting in a more robust fibre network. Furthermore, the beating would be attributed to fibre straightening, which improves fibre bonding and contributes to good paper strength. Overall, mechanical pulping combined with alkaline pre-treatment is an ideal technique for producing paper for grass-type biomass as compared to chemical pulping. Chemical pulping may be more suitable for making pulp from wood, however, from this study, it was too harsh to be applied on grass-type biomasses which are lower in lignin and extractive content. The high removal of lignin, whilst also maintaining hemicellulose content via the mechanical pulping process, has a significant impact on the surface characteristics of the laboratory paper handsheet derived from Napier grass and sugarcane stem. The high hemicellulose content of sugarcane-facilitated fibrillation will enormously improve the accessible surface area, fibre–fibre bonding, and eventually, hand-sheet mechanics. This work establishes a solid foundation of knowledge regarding the actual potential of grass-type biomass for paper production and presents an efficacious method for improving the conventional wood-pulping method.

## Figures and Tables

**Table 1:** Pulping condition for the Napier grass and sugarcane.

Pulping Condition	Chemical Pulping
Weight of sample	300 g
Sulphidity	25.0%
Active alkali 17%	17%
Fibre: liquor	1:7
Temperature during cooking	170 °C
Time to maximum temperature	60 min
Time at maximum temperature	120 min

**Table 2:** Chemical composition analysis of biomass and pulp obtained.

Type of Biomass	Pulping Technique	Lignin (%)	α-Cellulose (%)	Hemicellulose (%)	Extractive (%)	Ash Content (%)
Napier grass	Raw Fibre	21.77 (0.07)	46.58 (0.23)	31.04 (0.05)	3.42 (0.00)	4.16 (0.10)
	Chemical Pulping	3.31 (0.01)	89.42 (0.16)	3.38 (0.12)	1.18 (0.01)	1.31 (0.00)
	Mechanical Pulping	11.21 (0.02)	73.97 (0.08)	11.76 (0.02)	1.98 (0.02)	2.56 (0.01)
Sugarcane	Raw Fibre	22.32 (0.00)	40.34 (0.02)	33.39 (0.01)	3.95 (0.00)	8.27 (0.00)
	Chemical Pulping	2.76 (0.01)	87.98 (0.01)	3.56 (0.00)	1.30 (0.01)	4.52 (0.01)
	Mechanical Pulping	9.67 (0.00)	74.09 (0.01)	12.92 (0.00)	2.34 (0.00)	2.81 (0.00)

Note: The values in parentheses are the standard deviation of the mean values.

**Table 3:** Mechanical properties of characterization of a laboratory paper handsheet derived from Napier grass and sugarcane stem.

Type of Biomass	Pulping Technique	Folding Test	Burst (Lbf/cm^2^)	Tensile Test (KNm/g)	Tear Index (Mn)
Napier Grass	Chemical Pulping	23 ^cd^	5.7169 ^d^	15.4624 ^c^	286.4880 ^d^
	Chemical and Beating	46 ^b^	7.1414 ^c^	28.5712 ^a^	396.8563 ^b^
	Mechanical Pulping	106 ^a^	8.3044 ^a^	19.3793 ^c^	363.9777 ^c^
Sugarcane	Chemical Pulping	8 ^e^	6.9662 ^c^	17.2697 ^c^	259.3740 ^d^
	Chemical and Beating	14 ^de^	7.7335 ^b^	23.7757 ^b^	426.9640 ^a^
	Mechanical Pulping	37 ^cd^	8.7850 ^a^	19.1720 ^c^	373.6127 ^bc^
*p*-value		<0.001	<0.001	<0.001	<0.001

Note: Means followed by the same letter (a–e) in the same column are not significantly different at *p* ≤ 0.05 according to Tukey’s multiple comparisons test.

## Data Availability

All relevant data are within the manuscript.
